# A qualitative study of active travel amongst commuters and older adults living in market towns

**DOI:** 10.1186/s12889-023-15573-3

**Published:** 2023-05-10

**Authors:** Patricia E. Jessiman, Rosie E. Rowe, Russell Jago

**Affiliations:** 1 Bristol Medical School, Department of Population Health Sciences, University of Bristol, Bristol, UK; 2 Public Health and Community Safety Directorate, Oxfordshire County Council, Oxfordshire, UK; 3 Applied Research Collaboration West (NIHR ARC West), The National Institute for Health Research, University Hospitals Bristol and Weston NHS Foundation Trust, Bristol, BS1 2NT UK; 4 Centre for Exercise, Nutrition and Health Sciences, School for Policy Studies, University of Bristol, Bristol, UK

**Keywords:** Active Travel, Physical activity, Older adults, Commuter

## Abstract

**Background:**

Being physically active is associated with better health, but rates of physical inactivity are high amongst adults in England. Active travel, defined as making routine journeys in physically active ways, has been identified as a potential solution. There is a lack of research into how modal shift towards active travel can be encouraged in market towns. The aims of the current study are to understand how new cycling and walking infrastructure and community activation projects might support modal shift to active travel amongst commuters and older adults in market towns.

**Methods:**

This was a qualitative study using focus groups, ‘go-along’ interviews, and in-depth interviews as the main methods of data collection. Thirty-three participants (12 commuters and 21 older adults) took part across six focus groups. Eight of these also completed a go-along interview (4 walking, 4 cycling). Data were analysed using the Framework method of thematic analysis.

**Results:**

Market towns have existing advantages for active travel, being relatively compact with most routine destinations within easy reach. The barriers to active travel faced by older adults and commuters in market towns are similar to those in cities; poor infrastructure remains the key barrier. Poorly maintained paths are hazardous for older pedestrians, and low-or-no lighting and lack of well-connected, delineated cycle routes deter both commuters and older adults. One factor which does appear qualitatively different to cities is participants’ perception that the social norms of cycling differ in market towns.

**Conclusions:**

Policies to promote active travel in market towns are most likely to be effective when they include measures targeted at both individual behaviour change and population level measures like large-scale infrastructure improvements. Initiatives to change the social norms around cycling may be required to increase active travel rates.

**Supplementary Information:**

The online version contains supplementary material available at 10.1186/s12889-023-15573-3.

## Background

Physical inactivity has been described as a global pandemic [1], with a recent estimate that 7% of all-cause and cardiovascular disease deaths worldwide are attributable to low levels of physical activity [2]. In the UK, physical inactivity contributes to 1 in 6 deaths [3]. Being physically active is associated with better health, including a reduced risk of mortality, heart disease, diabetes and depression [3, 4]. The World Health Organisation (WHO) recommends at least 150 minutes of moderate-intensity physical activity per week for adults, a recommendation that is supported by the Chief Medical Officers of the UK [5, 6]. Currently only 61% of people in England aged 16 and over meet these guidelines [7].

Active travel, defined as making routine journeys in physically active ways, has been identified as a potential solution to high rates of inactivity. Walking or cycling for incidental journeys may be more easily incorporated into everyday life than undertaking exercise specifically designed to increase physical activity [6, 8]. The WHO have suggested that the benefits of active travel fall into three broad categories: mobility (e.g. reduced congestion, lower journey costs, more resilient transport systems), environmental (e.g. better air quality, reduced pollution), as well as health (e.g. cardiovascular health, mental health, physical fitness) [9]. The promotion of active travel became a clear UK Government priority under Boris Johnson’s administration with the publication of Gear Change [10] and the establishment of Active Travel England in 2022 as an executive agency responsible for making walking, wheeling (e.g. wheelchair and mobility scooter users) and cycling the preferred choice for everyone to get round in England. The UK Department for Transport’s latest Cycling and Walking Investment Strategy sets out the ambition that “*Cycling and walking will be the natural first choice for many journeys with half of all journeys in towns and cities being cycled or walked by 2030*” [11]. This will require significant modal shift. Data from the 2021 National Travel Survey in England reveal that 25% of travel trips made were under one mile, and the majority of these were made by walking. However the car remains by far the most common mode of travel for other trip lengths, even though most of these (72%) are under five miles [12]. The average person in England made 31% of all their trips by walking in 2021, with an average trip length of 0.8 miles. Cycle rates are lower still at 2% of all trips made, with an average trip length of 3.6 miles [13].

Active travel options may be unattractive because of habitual car dependence, real and perceived risks to safety, poor infrastructure for walking and cycling, and concerns about increased journey time and inconvenience [14, 15]. When asked what would encourage more walking trips, respondents in the 2021 National Travel Attitudes Study of individuals aged 16 and over in England favoured well maintained pavements, more direct walking routes, better provision for health needs (benches, toilets and access ramps), and safer roads and crossing points. Factors perceived to encourage more cycling included off-road and segregated cycle paths, safer roads, well-maintained road surfaces and more direct cycle routes [16].

The county of Oxfordshire centres around the city of Oxford in the south of England and includes rural areas and several small market towns. Market towns are towns in a rural areas which hold a regular public market, with a population of 2,000-20,000 residents, although given the expansion of many in England ‘larger market towns’ may have a population of up to 30,000 [17]. Promotion of active travel is a local policy priority for Oxfordshire County Council (OCC), which set out its ambition to increase rates of walking and cycling in the Local Transport and Connectivity Plan, formally adopted in July 2022 [18]. It outlines a vision to deliver a net-zero Oxfordshire transport and travel system by 2050 that enables the county to thrive while protecting the environment and making Oxfordshire a better place to live for all residents. It seeks to deliver this by: reducing the need to travel; discouraging individual private vehicle journeys; and making walking, cycling, public and shared transport the natural first choice. OCC also published its Active Travel Strategy [19] in July 2022 which sets out how it aims to increase walking, wheeling and cycling over the next 10 years. The strategy sets a county-wide target to increase the number of cycle trips to 1 million per week by 2031, from a current level of 600,000 cycle trips per week. Increasing levels of active travel in the county’s market towns is identified as playing an important part in delivering this objective.

OCC recently implemented a programme of interventions to encourage cycling and walking. The geographical areas targeted by these interventions include the market towns of Witney and Bicester, with population levels of 26,800 and 35,600 respectively [20]. Infrastructure improvements in Bicester include a new off-road cycling route and new cycle lanes into the town centre, wayfinding signs and speed limit reduction to 20mph on some central roads. In Witney changes include a new off-road shared cycle and pedestrian path, wider refuge islands, painted advisory cycle lanes on roads, wayfinding signs and speed limit reductions. These infrastructure changes are complimented by a range of community activation projects designed in partnership with local community groups to encourage modal shift to active travel. These include bike loan schemes, group walks and cycles, cycle training, and events to promote walking and cycling. The overall aims of the programme are to reduce congestion, address environmental issues, and improve population health and wellbeing. A logic model for the interventions is shown in Appendix 1.

Modal shift towards active travel is well-researched in cities and larger towns in England [8, 21–23] but there is a lack of research into how modal shift can be encouraged in smaller towns where infrastructure and public transport may be less well developed. In recent decades market towns have changed from being mere rural market and service centres to multifunctional areas of employment, services and visitor attractions, and home to a diverse range of residents with varying needs, including retired adults and working-aged adults who ‘commute out’ to other areas daily [24]. Local authority officers in Oxfordshire had implemented an evaluation of the impact of the active travel interventions that focussed on children and schools-based travel. However they were also interested to understand the impact of active travel interventions on two other population groups, commuters and older adults (aged over 65 years), and to determine whether interventions implemented in large, urban areas could be successfully replicated in market towns.

Older adults may experience greater overall health benefits from modal shift to active travel than younger adults [25]. The benefits of increased physical activity in older age include lower incidence of dementia, depression, heart disease, and other serious conditions [26], with cycling and walking especially linked with a reduced risk of all-cause mortality [25, 27]. Older adults’ decisions around active travel are more likely to be influenced by the physical environment [25]. The most important neighbourhood feature is the availability, accessibility and appeal of destinations – shops, food outlets, recreation and leisure activities and other services need to be an achievable distance for older adults to walk or cycle [28]. Research has shown a positive association between objectively assessed neighbourhood walkability (residential density, street connectivity, access to destinations and land use mix) and older adults walking for transport [28, 29]. Infrastructure is important too, for example well-maintained footpaths, availability of benches and the presence of streetlights. Older cyclists are concerned about safety, and prefer well-delineated cycle paths, separated from traffic, wide and obstacle free with safe road crossings [30, 31]. In the UK, an observational study found that older cyclists were more prevalent on cycling infrastructure that was separated from motorised traffic [32]. They may also be more likely than other age groups to deviate from the shortest route to avoid hills and obstacles [33].

Active commuting is beneficial for health. One UK study of over 150,000 adults found commuting by cycle was associated with a decreased risk of all-cause mortality, and death from cancer or cardiovascular disease, while walking to work was associated with decreased risk of cardiovascular disease [34]. Using active travel methods to commute is associated with sedentary job-types, shorter commuting distance and a lack of free car parking [35]. Interventions to promote active commuting have proved challenging in the UK. The iConnect study evaluated the effects of new walking and cycling routes at three sites in the UK. Although the aim of the new infrastructure was to transform ‘everyday’ journeys, residents were more likely to report using the routes for recreation and leisure purposes. This may have been because of poor connectivity [21]. More success has been seen with the ‘mini-Holland’ schemes in London, where an integrated approach includes infrastructure changes and redesigned town centres, cycle hubs at tube and rail stations, traffic calming in residential areas, and physically protected cycle lanes. Early evaluation findings show ‘mini-Holland’ areas are associated with increased use of active travel [8]. Research on attitudes towards cycling to work in the UK suggest non-cyclists are a heterogeneous group, including; those who would never contemplate cycling; those who would contemplate it but structural barriers such as hills and poor weather, or personal commitments dissuaded them; and those who occasionally cycle for whom positive feedback and social support may encourage more regular active commuting [36].

The aims of the current study are to understand how new cycling and walking infrastructure and community activation projects might support modal shift to active travel amongst commuters and older adults making within-town journeys in the market towns of Witney and Bicester. The research questions are:

1. How do commuters and older adults in market towns currently perceive active travel?
2. Do the infrastructure changes and community-based activities implemented across Witney and Bicester promote active travel amongst commuters and older adults?
3. What key steps or additional activities might be taken to increase modal shift and address the perceived barriers to active travel?

## Methods

This was a qualitative study using focus groups, ‘go-along’ interviews, and in-depth interviews as the main methods of data collection. Qualitative methods are ideal for this type of study as they are well-suited to explore in-depth, contextually-bound details for which quantitative approaches (e.g. population surveys) are insufficient, and can support the development and refinement of research findings (from inductive and interpretive stances) that are grounded in the experience, knowledge and perceptions of those living in the contexts of interest [37]. Data collection occurred between May and October 2022.

Prior to all data collection activities, participants were sent a detailed information sheet (PIS) detailing the aims of the study, funding, information about confidentiality and use of data, and reporting. Signed informed consent was provided by all participants and reiterated verbally at the start of data collection events. The study was granted ethical approval by the University of Bristol’s Faculty of Health Science Research Ethics Committee (Ref: 10144). The study protocol, developed in partnership with staff from OCC Public Health, Communities, and Transport teams, and Active Oxfordshire, is available elsewhere (https://fundingawards.nihr.ac.uk/award/NIHR135407).

### Public Involvement and Engagement

The study team coordinated an online consultation event with community stakeholders during study development, attended by a range of people living and working in Bicester and Witney with knowledge of the local area and the active travel interventions. This included staff from the district councils responsible for physical activity, and Health Walks; district and town councillors, the cycle champion and those working with community bike projects. The event was organised in partnership with Active Oxfordshire. The logic model for the active travel interventions (Appendix 1), draft research aims and objectives and proposed methodology for the study were discussed with participants and revised in response to their feedback. In addition, three members of the public, recruited through membership and user-group lists from Active Oxfordshire, were recruited to participate in a Public Advisory Panel, which met online seven times throughout the study. Panel members, who were all residents of Witney or Bicester, advised on all aspects of the study, including recruitment methods and materials, topic guides, early findings and outputs. Finally draft findings of the study were shared with members of the Oxfordshire Active Travel Roundtable, comprised of local councillors, residents, local authority staff and active travel campaigners. Around 60 people attended the online event in November 2022 and commented on early findings.

### Sampling and recruitment

The study team aimed to recruit residents living in Witney and Bicester from two target population groups:

1. Older adults (65-75years) who live independently and never, or only occasionally, use active travel methods for transportation, and
2. Working adults who commute to work at least three days per week who never, or occasionally, use active travel methods to commute to work (irrespective of use of cycling and walking for recreation).

The age-range for older adults was determined by the current UK retirement age of 65 years (at which people can receive the state pension in England), and an upper limit of 75 years beyond which transition to active travel may be less likely due to increasing rates of immobility and health concerns. Data from the UK National Travel Survey 2021 show a decline in the number and distance of walking trips per year from those in their 70s compared to those aged 60-69 years, and a decline in walking distance for the same ages [38]. Participants were recruited in the first instance to take part in a focus group, and also informed that they may be asked to take part in other aspects of the study (go-along interviews) although this was discretionary; participants could agree to only take part in the focus group.

Recruitment began in March 2022. Recruitment methods included placing physical posters about the study around the geographical areas of interest, including on noticeboards in community and leisure centres, public libraries, supermarkets, civic centres, churches, and shops. Emails with information about the study were sent to organisations targeted towards both population groups, for example large employers for the commuter population, and voluntary organisations, clubs and services for older adults. Adverts were also placed in local newspapers serving both towns. Recruitment was challenging and required repeated efforts in all these approaches. In May 2022, the study was adopted by the National Institute for Health Research Clinical Research Network (CRN). Specifically, the Thames Valley and South Midlands CRN supported recruitment through leafleting the residential areas of interest, social media adverts (Facebook) targeted at Witney and Bicester, as well as facilitating new items about the study on local newspapers and radio stations. In all cases posters, leaflets and other advertising material contained the lead researcher’s contact details with an invitation to contact her for more information. The full study PIS was sent to those who contacted her as well as the opportunity to ask further questions about the study before agreeing to take part.

Participants in go-along interviews were recruited from the focus group sample. Information about these were shared verbally by the researcher at the end of each group, and a detailed PIS shared with participants. Again, participants were invited to contact the researcher by email if they wanted to take part. We aimed to recruit five residents from each town; in the event that more expressed interest we planned to select participants to ensure a range of population-type and preferred travel mode (walking or cycling).

### Focus groups

Focus groups play an important role in health research, generating data through group interaction to support shared and contextualised knowledge, perceptions and experiences. They support the voicing of a range of views, which may coalesce around a shared opinion or conversely illustrate the polarity and diversity of views – both are possible in a well-moderated group [39]. Focus groups took place in community settings in the centre of each town and lasted one hour. A topic guide was developed that covered the four research questions, specified to the town in which the participants’ lived. The groups were facilitated by the lead researcher and digitally recorded (following verbal and written consent). Participants were given a £30 gift voucher as a thank you for their time and input.

### Go-along interviews

‘Go-along’ interviews are an established method for exploring the implications of place for health and wellbeing and active travel methods in particular, including amongst older adults [30, 31, 40]. The term refers to a qualitative interview that is conducted during, or shortly after, an accompanied journey in the participants’ neighbourhood by car (ride along), bicycle (bike-along) or foot (walk-along). The researcher asks about participants’ experiences, interpretations, and practices whilst in the local environment and undertaking the same journey. This allows the combination of two qualitative data collection techniques: observation and interview, building on their strengths and reducing limitations. The researcher is able to observe and acclimatise herself with an environment and make observations that participants (locals) may miss; but also better understand participant perception and experiences through questions. Interviews ‘in the moment’ also support rapport building and reduce power dynamics as the participant is the ‘guide’ . We adapted the methodology used by Van Cauwenberg and colleagues [31] to suit the needs of older adults and working age commuters, and for either walking or cycling journeys. The following steps were followed:

1. Participants completed a short questionnaire which included questions on: demographics; preferred mode of transportation and why; experience of active travel methods; and an assessment of the neighbourhood walkability/cycle-ability (using an adapted version of the Neighbourhood Environment Walkability Scale (NEWS) [41] to determine distance to routine destinations. The NEWS was developed in the US and is a popular measure of perception of neighbourhood environment. Several versions exist [42, 43] and we adapted the questionnaire with our public panel members to suit the needs of UK commuters and older adults (see Appendix 2).
2. Participants agreed a date and time, and mode (cycling or walking) for the go-along journey with the researcher. All journeys took between 40 and 60 minutes.
3. During the journey, the participant was invited to vocalise their thoughts and perceptions of things in the environment that made the trip more or less comfortable, enjoyable, safe and convenient. This was prompted by questions from the researcher where it was safe to do so. The journey was audibly and visually recorded using a camera (GoPro HERO10) which participants wore on an outward facing chest harness.
4. Video images and voice recordings were used to inform a follow-up interview shortly after the accompanied journey. Participants were asked to view some of the images and compliment their statements made during the journey to explore in more detail their perceptions of active travel. Interviews could take place in person immediately after the go-along, or online, dependant of participants’ preference. Interviews lasted between 30 and 45 minutes.

The researcher also made detailed field notes immediately after the journey. Participants were given a £50 voucher for their time. Visual footage from the go-along interviews were not analysed, but used as a participatory prompt for interviews. Participants were made aware that the visual and audio recording from the go-along would not be shared beyond the research team, and be securely deleted soon after the follow-up interview. Still images from the recordings which did not contain any identifiable information were used with consent to illustrate some findings, particularly around infrastructure.

### Analysis

Focus groups, interview data and voice recordings from go-along interviews were fully transcribed. We used the Framework method of thematic analysis [44, 45]. Following a review of the transcripts, the lead researcher developed a draft conceptual framework that included the key themes and sub-themes identified in the data for each target population (older adults and commuters). The thematic framework was driven by the data, but also informed by our research questions. The thematic framework was reviewed by another member of the research team against a sample of transcripts to ensure a correct ’fit’ with the data and revised until agreement was reached. The frameworks were used to code the data, assigning both verbatim and summarised excerpts of the transcript to one or more theme or sub-theme.

A systematic approach to data management was adopted, coding the transcripts into the framework using NVivo software (https://www.qsrinternational.com/nvivo-qualitative-data-analysis-software/home) . A framework matrix was developed in NVivo, using the themes as column headings and data transcripts as rows. The matrix cells were populated with verbatim and summarised data from the transcripts, as well as analytical notes made by the researcher (‘charting’). Once all the transcripts were charted, the analysis continued using the Framework matrix as a detailed and accessible overview of the data populating each theme and sub-theme from every data collection event. Framework matrices make possible the capacity to explore the dataset through themes and subthemes, and by respondent type. A summary of the data under each subtheme was developed to inform the next stage of the analysis, moving up the analytical hierarchy to explore patterns and associations between themes in the data.

## Results

Thirty-three participants took part across six focus groups for the study (Table 1). The groups were targeted to either commuters (N=12) or older adults (N=21) in each town, and ranged between three and nine participants. All participants self-reported their ethnicity as White British with the exception of one White USA and another from the Indian subcontinent. There was an equal gender split, with one older adult not declaring gender.

**Table 1:** Focus group sample

**Focus group**	**Town**	**Participant Type**	**N**
1	Witney	Commuters	3
2	Witney	Commuters	3
3	Witney	Older adults	5
4	Witney	Older adults	7
5	Bicester	Commuters	6
6	Bicester	Older adults	9

Eight went on to complete a go-along interview. Again, there was an equal gender split and an equal split across travel mode (cycling or walking). Five took place in Witney. Four participants were part of the commuter sample, one of whom was also in the older adult group (and took part in a focus group aimed at older adults). Full details can be seen in Table 2.

**Table 2:** Go-along sample and travel mode

**Go along**	**Town**	**Participant Type**	**Gender**	**Mode**
1	Witney	Older adult/commuter	M	Walk
2	Witney	Older adult	M	Cycle
3	Witney	Commuter	F	Cycle
4	Witney	Commuter	F	Walk
5	Bicester	Older adult	M	Cycle
6	Bicester	Older adult	M	Cycle
7	Bicester	Older adult	F	Walk
8	Witney	Commuter	F	Walk

### RQ1: How do commuters and older adults in market towns currently perceive active travel?

Both the market towns in the study have relatively compact centres with newer housing estates built on the outskirts. The eight participants who completed the adapted Walkability Scale reported that most routine destinations such as shops and services were within a 20-minute walk from home, with a small number slightly further (most usually a pharmacy, secondary school, large supermarket and bank) but still reachable on foot in under 30 minutes. The exception was walking time to a train station in Witney, which does not have one. Respondents in focus groups agreed that most destinations were within walking distance and that both towns were pleasant to walk around. Some Bicester participants reported that services such as GP practices and large shops were increasingly moving to the outskirts of the town, ‘hollowing out’ the centre and making active travel less convenient. Many cited aspects of the towns perceived to promote active travel, in particular that the towns were mostly flat, there were prominent signs showing cycle and walking journey times to destinations, and numerous cycle and walking paths (Appendix 3 Pictures 1-2). These latter were often shared paths, and particularly prevalent around the newer estates on the outskirts.

### Older adults

Older adults were more likely to report using active travel methods for within-town trips than those making commuter journeys. Reasons given for choosing active travel included inability to drive or access a car, quicker journey times (avoiding traffic congestion) and enjoying a pleasant route. Health was frequently cited as a motivator for active travel. Some had increased their walking and/or cycling trips as a means of maintaining good health and fitness in later life and avoiding immobility, or in response to a recent diagnosis of ill-health or loss of eyesight.

*If you asked me to give a ranking order of why I cycle, health is always going to be the number one. The time to get down the town is important to me. There’s a social aspect to it, I’ve met lots of people through cycling. But to go back to health, that is the case, and it remains the number-one reason why I cycle.*

GO2 older adult (M)

While making within-town trips on foot was reported as relatively easy and pleasant, older adults were more likely than commuters to have concerns about walking. Negotiating hills was raised as a problem, and the additional time taken for trips when compared to the car. Cars were also the preferred travel mode for weekly shopping trips and other tasks that involved carrying bulky or heavy items. The remaining disadvantages for older adults centred around infrastructure. The most prominent complaint was poor maintenance of paths and pavements, including uneven surfaces increasing the risk of trips and falls, and overgrowth from bordering trees and bushes (see Appendix 3 Pictures 3-5).

*You have to walk in the road because the hedgerows are just never kept back. Anyone with any disability would struggle down there. …[]…I mean, I'd love to be able to walk every day, but I just feel it's not safe to do so.*

Older adult (M)

Overgrowth and poor (or no) lighting on footpaths also made them unattractive, when poor visibility made tripping or falling more likely, and concern for personal safety from other path users prevented most older adults from walking after dark or in the winter months. Paths shared with cyclists were often perceived as unpleasant for older pedestrians because of the risk of collisions. Participants noted that while most cyclists were considerate, some would cycle too fast or recklessly, making walking the paths feel dangerous. A smaller number of older adults also reported these worries about pavement cyclists.

*It’s the conflict with cyclists actually. I’m sorry to say. Four times in the last year I’ve had close scrapes, where either- once I was injured, but that was a child just tearing round the corner, that just happened. Two or three times it’s adults on pavements that are fairly narrow, and one I actually had an argument with because he said, “It’s not illegal, cycling on the pavement and so on.” Well, it is, actually.*

Older adult (M)

Older adults perceived that more pedestrian crossings, particularly on busy roads in both towns, would make walking easier. In Witney, respondents noted during go-alongs that most changed quickly, avoiding long waits to cross the road. However in Bicester, long crossing times were raised during the focus groups and in all three go-along interviews. Residents walking to some of the newer housing estates and one of the larger supermarkets have to cross a major multi-carriage route joining the town with the nearby motorway. Pedestrian light-controlled crossings have long wait times; during one go-along journey it took over 2.5 minutes to cross waiting for the pedestrian signal and there are several crossings to negotiate. Participants note this encourages ‘risky’ crossings, or puts them off walking round there completely. Bicester residents also noted that the road prevents easy access to a new retail park as one side remains unpaved (Appendix 3 Picture 6-7).

*So [the supermarket's] almost impossible to get to on foot, or it's dangerous. The same with cycling, it's dangerous. So I do tend to drive to [the supermarket]*.

Older adult (M)

*There is no path towards the new retail park - just a dirt track, even though retail park has been open for three years. When they built that retail park, the council in their wisdom decided that it was quite acceptable for people to walk all the way along on the opposite side of the road, cross four lanes of traffic, wait in the middle, cross another three lanes of traffic then walk back on yourself to get to that retail park. Hardly anybody does that, so the dirt path is just a dirt path now and people do take risks crossing the road where there isn't a crossing.*

GO7 older adult (F)

Older adults who rarely or never cycled gave several reasons why not. Again, hills were a worry, and bad weather more of a concern when cycling than walking because of the perceived added danger. Some older adults, mostly female, also expressed a lack of self-efficacy as cyclists, stating ‘*I’m too old’* or ‘*too wobbly’.* For some this lack of confidence was the result of previous accidents.

*I always cycled, but the path, it wasn’t a proper cycle path, and it was too narrow. And anyway, I fell off and fractured my pelvis, so I was shot off to hospital for that.*

Older adult (F)

Many of the perceived barriers to cycling for older adults were about poor infrastructure. Some reported feeling too unsafe to cycle on roads, but a lack of off-road cycle paths from door to destination meant they would not cycle for routine trips. Cycling on roads presented a number of problems, including being caught up in congested traffic, breathing in fumes, dealing with narrow streets which led to either ‘close passes’ from motorised traffic, or a perception that the cyclist was holding up traffic and frustrating drivers.

*When there’s a build-up of traffic behind me, I pull over. That’s not [me] doing his good deed for the day, or perhaps I should say it is, it’s actually [me] being aware that the people behind me by now are getting frustrated, and that puts me at risk.*

Older adult (M)

In one new housing estate in Bicester, traffic calming measures built into the design include high kerbs, narrow carriageways and green barriers between carriageways to deter overtaking. These measures deterred some older adults from cycling because of insufficient opportunity for vehicles to pass cyclists, leading to a perception that they are at increased risk.

*So I don’t cycle that way. I have to drive to play football now. There's only one person who dares to cycle on that road. The vans are in such a rush delivering their Amazon stuff that they squeeze past you. So I don’t- that’s another reason I don’t cycle.*

Older adult (M)

Dedicated, off-road cycle paths were preferred to using the road network. However where these existed in the market towns they were often poorly lit and maintained; again paths overgrown with vegetation, or too dark in the evenings or in winter, would not be used by most older adults.

### Commuters

Participants in the commuting groups reported far less disadvantages about walking than older adults, though female participants shared the concern about poorly lit paths. These were particularly prominent in newer housing estates, and several women who lived in these reported the paths unusable at night, forcing them to drive or walk much longer distances by road to reach their homes.

*There’s a section of path if you get into [my estate], after the new crossing, walk in a bit and then you have to go down and then back up. And this sort of down and back up – and everybody knows I’m talking about – it’s not lit. …[]…it’s trees and it’s dark, so I won't go there. As soon as it’s even dusky, I just feel nervous, and so I won't do it. So then I think well the only alternative is walking all the way round, or I’ll drive.*

GO8 commuter (F)

Many of the commuters’ concerns about cycling were shared with those of older adults: poor infrastructure and concerns about personal safety. Commuters worried less about hills and weather than older adults but shared many of the same concerns about the dangers of cycling on roads and unlit paths, and poor connectivity of dedicated cycle paths in the towns. Painted advisory cycle lanes on roads were not perceived as helpful, as often the roads were too narrow to allow safe passing distance for vehicles and were often blocked by parked cars (Appendix 3 Picture 8). Those who did cycle were appreciative of Advanced Stop Lines, (bike boxes) at junctions, particularly when turning right. Female respondents in particular reported concerns for their personal safety cycling after dark, and getting into conflict with drivers.

*For me, there’s an added thing of if you’re out and about at night, I wouldn't want to cycle on my own. I don’t have somebody at home, so if I’m aware that I’m going to be on my own and coming back late, then I will take the car, which I’m annoyed with. I shouldn't feel like that.*

Commuter (F)

*At some point there’s a big queue of traffic. I feel pressure that the cars are thinking, “You’re not a car, get over to the side, I’m going to overtake you even though there is nowhere to overtake.” You’re just going to squish me into a box and I’ll have to…Often I just get off the bike and walk. I think part of it is that I’m a woman as well, I honestly do. A male car driver won’t see that I’m in charge.*

GO3 commuter (F)

Cycle paths shared with pedestrians were perceived as better than road cycling for most respondents, although these are slower for cyclists because of the need to take care around walkers, and the additional road crossings at junctions. Commuters were less likely to use these for work journeys. The additional time required to prepare for cycling (ensuring the bike is road-safe; wearing waterproof and high visibility clothing etc) was an additional barrier.

*Walking, you just get out and you walk, but cycling you’ve got to get the bike out the garage, you’ve got to find all the stuff, make sure the tyres are pumped up etc. It’s just a lot more hassle.*

Commuter (F)

Respondents worried about security of bikes and accessories when left outside the home. This was also raised by older adults, but was a particular issue for commuters who may have to leave a bike outside the workplace or at a train or bus station for long periods of time. There was a general perception that bike theft was not prioritised by the police, and a lack of secure, safe locking points around both towns and at public transport hubs. In some cases, concern about bike theft was deterring the purchase of electric bikes, which could help resolve some of the issues around negotiating hills and delaying traffic.

Finally, several respondents noted that cycling was a less common activity in market towns than in larger cities. This meant they believed local drivers were less ‘cyclist-aware’, and fewer cyclists on the road meant there was no safety in numbers. The social norms around cycling were perceived as different between cities and small towns, which made cycling less attractive.

*Road users hate us, hate cyclists. Two cyclists together is probably enough for some drivers to just think, “What’s this town turned to, a bunch of cyclists.” …[]…I feel less safe cycling in Witney than I used to in Oxford. That feels a bit weird. It shouldn’t feel that way.*

Commuter (F)

### RQ2. Do the infrastructure changes and community-based activities implemented across Witney and Bicester promote active travel amongst commuters and older adults?

Participants were asked during the focus groups about the infrastructure changes and community activation projects implemented by the local authority. There was no discernible difference between the two population groups and as such findings for both groups are combined in this section. Off-road shared paths for cyclists and pedestrians were broadly welcomed, with the proviso mentioned above that cyclists were encouraged to ‘share with care’ to avoid collisions with pedestrians. New signs around both towns with cycling and walking times to popular destinations were perceived as useful way to promote active travel.

Painted cycle paths on roads were less valued, as respondents believed they were often ignored by drivers, frequently parked on, and could cause conflict where lanes merged:

*We’d all like a physical separation between bicycle and motor vehicle. And I also recognise that this is a medieval town, so room is at a premium. Now this particular part of this* [painted*] cycle lane is better than nothing. Behind where I am there, the cycle lane and the lane for traffic merge, and nobody knows who has got right of way. And that leads to confusion. It certainly leads to confrontation, as well. So in some ways, I wish...where the two lanes merge, it’s almost better not having it. Because I, as a cyclist, am absolutely certain in my mind that I’ve got right of way. But so is that motorist behind me.*

GO1 (M)

Respondents supported prioritising cyclists and walkers at junctions, either through ‘bike boxes’ at the front of traffic lanes, or ensuring that crossing points did not have too long a wait for walkers (and where applicable cyclists) to cross. Despite efforts to increase cycle routes in both towns, lack of connectivity of paths remains an issue.

*End to end cycle paths …[]… even on my route, I think it’s nice and straightforward, I’ve got a lovely cycle path a lot of the way, but for some of the way there’s just nothing, and it’s forcing you onto the road.*

Commuter (M)

Reduced speed limits in residential areas were supported by most participants, though many believed that they were not enforced and drivers could speed with impunity. Enforcement of the law emerged as a key concern for many, including of speeding and unsafe driving, and reckless cycling on shared paths with pedestrians.

The community activation projects were perceived as helpful and encouraging ways to promote active travel. A small number of both commuters and older adults had participated in these, either through a bike loan, going on a guided walk, or using the cycle training schemes. Where respondents had participated they were very positive about these types of approaches:

*I consider myself a novice rider so I don’t like riding on roads. But I joined the group* [Bike User group]*, and they've given me a biking buddy who's going to teach me, guide me along. We'll go on cycle rides together, and that’s just what I need, just to get used to the roads.*

Older adult (F)

*The group health walks I did. They start at one of the practices, the doctor’s practice. They take you different- I didn’t realise there were so many walks you could do round here. I was absolutely flabbergasted. Very good.*

Older adult (F)

*I worked at the NHS and during COVID they set up this wonderful scheme, […] Bike Project, which I have had the best experience from, so, so nice. Because I work for the NHS, they said people who work for the NHS during COVID – because we carried on working – were entitled to a free bike, which was amazing. I have Rosie. I’ve named her. She’s red. She’s lovely. A little bit of rust, but it's incredible.*

Commuter (F)

However, the majority of respondents had not heard of many of these activation projects until they were mentioned during the focus groups. While perceived as useful ideas, respondents would welcome wider promotion and awareness of walking and cycling promotion schemes in both towns.

### RQ3. What key steps or additional activities might be taken to increase modal shift and address the perceived barriers to active travel?

Both groups of respondents advocated for better maintenance of paths for walking and cycling in both towns, including addressing uneven surfaces, overgrown vegetation, and litter (including broken glass). Adequate lighting is required for these paths to remain accessible for most respondents, but especially females, after dark. Most respondents wanted more, and better, signage to encourage use of the correct side of the path where it was split (not all paths are). For older adults using these paths to walk, this was perceived to reduce the incidence of falls, and collisions with cyclists. This was especially important to older pedestrians who worried about the risks of falls or collisions. Commuters wanted these signs to remind pedestrians that cyclists had a right to use the path.

*I also think they’ve got to maintain the pavements a lot better, because that’s some of the reason why suddenly as a pedestrian you might suddenly veer. Somebody thinks, "Oh, you're going just a small way where you live," but there's a broken paving slab.*

Older adult (M)

*There are tiny little bike signs there, you know, this is still a cycle path as well, but it’s really easy to miss, and so all the pedestrians get really funny with you for cycling on that bit. I’m allowed to. I’m going quite slow now to be polite, but you will get people kind of really trying to block your way.*

Commuter (F)

Infrastructure improvements that to date have not been addressed by the local authority include improved bike security and access to bike lockers at key destinations like public transport hubs and town centres. Better provision for bike security was reported as a necessary requirement for some respondents to travel by bike and in some cases, invest in an e-bike.

*The lack of security puts me off getting an electric bike. I cycle on a really old bike though. I don’t expect it to get nicked, but I am thinking about getting an electric bike. That worries me.*

Commuter (M)

*Yes, because I chain both the frame to something structural, you know, like, a cycle pole, and also I chain the back wheel, because that’s where the motor is, so that would be the bit that would be most valuable to steal. So, I am slightly conscious that if I went to the local station...[]and I would have to leave it all day.*

Older adult (M)

More generally, respondents believed that the most useful means to promote active travel was through initiatives that encouraged and rewarded it, rather than punished residents for driving cars. Respondents wanted better awareness of community active travel projects and wider education and promotion of the positive benefits of active travel, including for health, personal finance, and the environment. Role modelling and promotion was thought to be especially important in smaller towns where active travel may be less normalised.

*You need people of all different colours, ages, being out there. You need to be seeing people actively travelling, people that look like you so you can relate and think, “Oh actually, I could be doing this.”*

Older adult (F)

Some respondents were keen to see more effort to promote a culture change in market towns, where active travel (in particular cycling) could be more normalised, encouraging a shift from feeling allowed to cycle, to feeling celebrated.

*Let’s celebrate* [cycling]*, let’s make it obvious. You’re not just saying, “Yes, yes, you’re allowed.” It’s, “You’re welcome. We think you’re doing a good job. Well done for not ruining the planet.”*

GO3 Commuter (F)

## Discussion

Active travel has been identified as a potential solution to high rates of physical inactivity in the UK, which currently contributes to 1 in 6 deaths [3]. The current study used qualitative methods to understand how infrastructure improvements and community activation projects implemented in market towns are perceived by older adults and commuters, who can experience health benefits of increased active travel [25–27, 34]. Creating a health-enabling built environment is an important mechanism whereby public health can address the wider determinants of health and support populations to maintain a healthy weight and reduce the risk of cardiovascular disease, type 2 diabetes, cancer and depression. The Health Foundation has identified that 1,189 early deaths in England could be prevented if the current level of walking and cycling rates increased to the highest average daily miles walked and cycled [46]. It explored what interventions might encourage modal shift amongst these two groups and address the perceived barriers to active travel. Active travel has been well researched in large urban areas, but less is known about how to encourage modal shift in smaller market towns and whether the perceived barriers and ways of addressing them are qualitatively different than in cities.

Market towns have existing advantages for active travel, being relatively compact with most routine destinations within easy reach. Respondents in our study reported most within-town trips to shops, services and other popular destinations were within a 20-minute walk from home, with the remainder walkable within 30 minutes. This suggests that market towns, at least the two in our study, are already near meeting the criteria for “20-minute neighbourhoods”, a design concept for urban development where daily services can be accessed within a 20-minute walk [47, 48]. Guidance on creating 20-minute neighbourhoods in England suggests that it may be easier to ensure that market towns meet these criteria than larger towns and neighbourhoods built in the 20^th^ century and designed to prioritise the car [49]. Perhaps because of these advantages, participants in our study who did report using active travel modes for within town trips did so because they found the towns pleasant to walk around, with active modes sometimes quicker than using the car. This is supported by other studies; a systematic review of the impact of the neighbourhood environment on active travel amongst older adults found that walking for transport was associated with neighbourhood walkability and availability of destinations [28] . Older adults were also motivated by health factors; either promoting and maintaining good health or in response to a recent poor diagnosis. The commuters in our study were frequently ‘commuting out’ of town to work, in most cases making active travel modes impracticable. Their motivation for active within-town trips included convenience (quicker or no parking issues), costs, and climate concerns.

Older adults reported a range of concerns about walking and cycling in market towns. Hills, poor weather, and self-efficacy were of concern, but the main barriers centred around infrastructure, in particular uneven paths and pavements, overgrown vegetation, and poor or no lighting. The importance of well-maintained footpaths to walking amongst older adults has been reported elsewhere [28]. Poor connectivity of cycle paths, preference for off-road delineated paths, worry about conflict with motorised traffic, and worry about theft were the main barriers to cycling. This is unsurprising as mobility and sensory impairments may impact older adult-cyclists more, and their preference for off-road paths, smooth and obstacle free, and safe crossings have been found in other studies [31, 32]. There were fewer concerns about walking, although shared paths with cyclists were considered unsafe by some and in Bicester in particular, long wait times at crossings were off-putting.

Commuters shared the concerns of older adults about poorly lit paths, particularly females. The prominence of dark paths in the newer housing estates surrounding market towns presents a significant barrier to both walking and cycling amongst women who live there. Commuters also shared older adults’ concerns about cycling on roads and potential conflict with motorised traffic – the so-called ‘hostile environments’ reported in other studies [21]. Again, there was some evidence of a gendered effect, with female respondents noting concerns about coming into conflict with male drivers. Female cyclists’ increased worry (compared to men) over being bullied or verbally abused has been reported elsewhere [50]. Shared paths with pedestrians are slower for cyclists than road cycling, which together with the perceived additional burdens of ‘getting ready’ to cycle presented another barrier for active commuting. Significant concerns about theft prevented some commuters from making at least part of their commuting journey using active modes because of a reluctance to leave cycles at train and bus stations. Theft concerns feature consistently in other studies of e-bike use [51–53].

Asked what would encourage more active travel in their towns, respondents were broadly supportive of more and better-connected off-road paths; where these were shared-use they wanted better signage to promote accessibility for both cyclists and pedestrians. Painted cycle paths on roads were not successful in encouraging respondents, who worry about holding up traffic, and conflict and collisions with vehicles when cycling on roads. Reduced traffic speeds may help with this but are not perceived to be enforced strongly enough. However, traffic calming and restrictive measures may be worth pursuing. A recent systematic review and meta-analysis of population-level active travel interventions found that while interventions with positive strategies (“Carrots” e.g. cycle training, improved walking and cycling infrastructure) were more likely to be evaluated, interventions that include “stick” elements (e.g. reduced car parking, traffic restrictions) may be more effective [54].

Maintenance of cycle and footpaths was seen as key, including smoothing out surfaces, cleaning them of litter and glass, and cutting back overgrown vegetation that too often narrowed existing paths. Lighting is also key to path accessibility after dark and during the winter months. Both older adults and commuters wanted more secure cycle parking, including bike lockers, in their towns. These findings are broadly supported by research conducted in UK cities and towns by Sustrans. Improved accessibility for pedestrians is also likely to improve access for ‘wheelers’; those using wheelchairs and/or mobility scooters [55, 56].

The community activation projects such as group walks and cycles, cycle training and buddy scheme implemented by OCC and Active Oxfordshire were welcomed by most respondents as useful ways of encouraging novice cyclists and reluctant pedestrians to try active travel. However, there was a lack of knowledge about these schemes. Better promotion of these types of interventions is necessary if commuters and older adults not already involved in walking and cycling are to engage in them. This matters; a recent systematic review of how policies contribute to increased physical activity concludes that while new infrastructure can be effective, policies are most effective when they are comprehensive and also include educational and promotion activity [57]. While infrastructure interventions may be most popular amongst our respondents, they may be beyond reach of cash-strapped local authorities or smaller organisations interested in supporting active travel. A review of smaller scale interventions to promote cycling at the micro-physical level (for example improved bike storage and signage) as well as at the individual and social level (e.g. training, loan schemes) suggests that these are important because the most effective means of promoting active travel are integrated and complementary measures targeted at all levels. Infrastructure works, but is not enough [27, 58].

One factor we identified which does appear qualitatively different to cities is participants’ perception of the social norms of cycling. Several reported that cycling was far less common in market towns, leading to the belief that drivers would be less ‘cyclist aware’ as a result, resulting in a greater risk of collisions and conflict. This perception is supported by studies examining the relationship between numbers of walkers and bicyclists, and the incidence of collisions, showing there *is* safety in numbers; the greater number of cyclists and walkers, the lower rate of collisions involving vehicles [59, 60]. Efforts to change the social norms about cycling in market towns, such as more education and promotional activities ‘celebrating’ cycling, may be worth pursuing.

### Strengths and limitations

The study is limited by a smaller sample size than we intended. We tried a range of recruitment strategies, and were supported by the Clinical Research Network, however we faced difficulties recruiting respondents currently not interested in walking or cycling to a qualitative study about active travel. We had a limited timescale for this study, and we would recommend any future similar studies plan for a long recruitment period. These difficulties also meant that we had to include some participants who walked or cycled on occasion or more regularly. This may impact on the generalisability of the findings for those older adults and commuters who never, or rarely use active travel modes, although we do note that behaviour change is a process (not an event) and that the findings may be useful to those seeking to encourage infrequent active travellers to do so more often [36]. All of the participants except one were White British; while this reflected the population demographics of both towns, it may further limit the generalisability of the findings to adults of different ethnicity.

The key strength of this study is the use of focus groups and go-along journeys, combining observation and interview, facilitating rich data grounded to the specific issues supporting or limiting active travel in each market town. We believe this is the first study that has explored attitudes towards active travel held by residents of market towns.

## Conclusion

Market towns have intrinsic advantages for active travel due to their compact nature but have received little attention in the active travel literature. This matters if the residents of market towns are to gain the significant positive health effects associated with active travel. This study suggests that the barriers faced by older adults and commuters in market towns are mostly no different to those in cities; poor infrastructure remains the key barrier. Poorly maintained paths are particularly hazardous for older pedestrians, and low-or-no lighting and lack of well-connected, delineated cycle routes deter both commuters and older adults. Like cities, policies to promote active travel in market towns are most likely to be effective when they include a suite of comprehensive, integrated and complementary measures targeted at a range of levels from individual behaviour change to population level measures like large-scale infrastructure improvements. Attitudes towards active travel, in particular cycling, may differ in market towns and initiatives to change the social norms around cycling may be required to increase active travel rates.

## Supplementary Information

**Additional file 1.**

**Additional file 2.**

**Additional file 3.**

## Data Availability

Anonymised qualitative transcripts used during the current study are available from the corresponding author on reasonable request.

## Abbreviations

CRN: Clinical research network
NEWS: Neighbourhood environment walkability scale
NIHR: National institute for health research
OCC: Oxfordshire county council
PIS: Participant information sheet
