# Supplementary material for: A qualitative study of active travel amongst commuters and older adults living in market towns
Source: BMC Public Health. 2023 May 10;23:840. doi: 10.1186/s12889-023-15573-3 (PMC10170734; doi:10.1186/s12889-023-15573-3)

**OCC Active Travel Logic Model**

**Intervention Focus:** Witney and Bicester; Active Oxfordshire interventions, infrastructure changes
**Target user group(s)**: commuters (in particular within-town commuting but also commute to train station etc for on-commuters) and older adults

Definitions of intervention functions and change mechanisms taken from the Behaviour Change Wheel (Michie et al 2011)

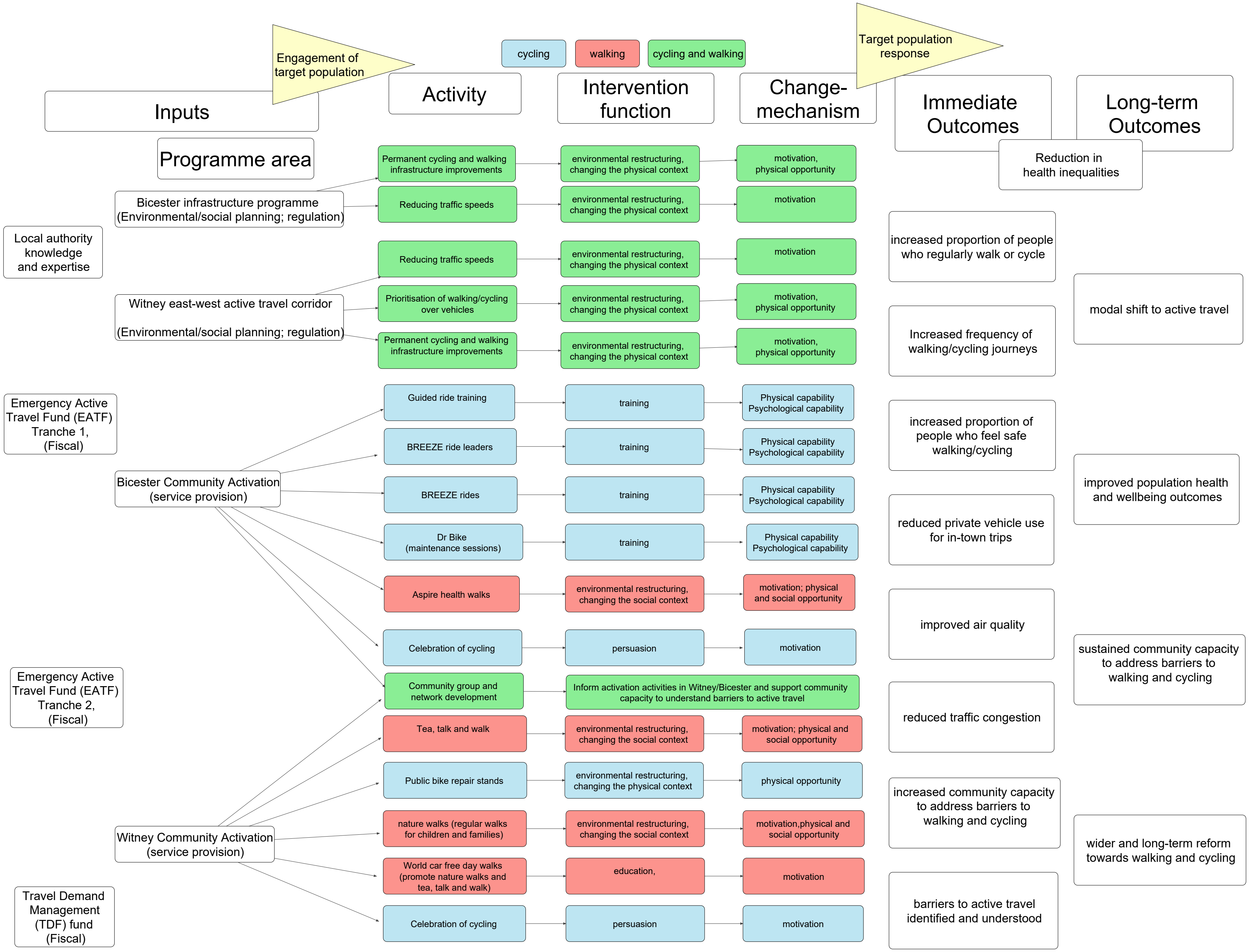

Supplement: Supplementary file 1 — Additional file 1. [file 12889_2023_15573_MOESM1_ESM.pdf]
