# Supplementary material for: A qualitative study of active travel amongst commuters and older adults living in market towns
Source: BMC Public Health. 2023 May 10;23:840. doi: 10.1186/s12889-023-15573-3 (PMC10170734; doi:10.1186/s12889-023-15573-3)
Supplement: Supplementary file 2 — Additional file 2. [file 12889_2023_15573_MOESM2_ESM.pdf]

Appendix 2. Pre Go-along Interview Questionnaire

Thank you for your interest in taking part in our research study about Active Travel in Witney and Bicester.

This study is being conducted by researchers from the University of Bristol. We are part of Bristol and Cardiff Public Health Intervention Responsive Studies Team (PHIRST), a programme funded by the National Institute for Health Research (NIHR).

The aim of the current study is to understand how new cycling and walking infrastructure and community activation projects might support active travel amongst **commuters** and **older adults** making within-town journeys in Witney and Bicester.

You are being asked to complete this questionnaire because you have consented to take part in a 'Go-Along' interview for the study. The questionnaire asks about your Active Travel, and your neighbourhood. The researcher will discuss your answers with you during the follow-up interview.

The questionnaire should take **no more than 15 minutes** to complete. If you are happy to continue, please proceed to the next page.

1. Name:____
2. Age:

	Please tick one:
Under 20 years	
20-29 years	
30-39 years	
40-49 years	
50-59 years	
60-69 years	
70+ years	

3. Gender:

	Please tick one:
Male	
Female	
Other (please specify)	
Prefer not to say	

4. The Equality Act (2010) defines a person as having a disability if he or she ‘has a physical or mental impairment that has a ‘substantial’ and ‘long term’ negative effect on your ability to do normal daily activities’. Do you consider yourself to have such a disability?

	Please tick one:
Yes	
No	
Prefer not to say	

5. Ethnicity :

	Please tick one:
**White**	
English, Welsh, Scottish, Northern Irish or British Irish	
Gypsy or Irish Traveller	
Any other White background	
**Mixed or Multiple ethnic groups**	
White and Black Caribbean	
White and Black African	
White and Asian	
Any other Mixed or Multiple ethnic background	
**Asian or Asian British**	
Indian	
Pakistani	
Bangladeshi	
Chinese	
Any other Asian Background	
**Black, African, Caribbean or Black British**	
African	
Caribbean	
Any other Black, African or Caribbean background	
**Other ethnic group**	
Arab	
Other ethnic group	
**Prefer not to say**	

We are going to ask some questions abouts Active Travel. By this we mean **walking** or **cycling**. Active travel is defined as incidental travel to reach a destination, e.g. going to work, or the shop. It is not about walking or cycling for recreation, pleasure, or leisure.

6. Please tell us your most preferred way to travel around within Witney

	Please tick one:
Car	
Motorbike	
Bus	
Train	
Cycle	
Walk	
Other (please specify)	

7. *Why* is this your preferred way to travel in Witney?

____

____

____

____

____

____

8. How often do you use **cycling** for active travel within Witney? (Remember, this means cycling to somewhere you need to be, and not cycling for leisure or recreation.)

Please tick one option.

	Please tick one:
Every day	
Most days	
Once or twice a week	
A few times a month	
Rarely	
Never	

9. And how often do you use **walking** for active travel within Witney?

Please tick one option.

	Please tick one:
Every day	
Most days	
Once or twice a week	
A few times a month	
Rarely	
Never	

10. What matters to you when deciding how to travel (e.g. choosing to take the car, bus, cycle or walk etc)

(Please tick one for each option below)

	Matters a lot	Matters a bit	Does not matter at all
Time of day			
Journey time			
Weather			
Comfort			
Convenience			
Personal safety			
Security (of belongings e.g. from theft)			
Who I am travelling with			
What I am wearing			
What I am carrying			
Traffic/congestion			
Ease of parking (car)			
Cost			

11. Do you take anything else into consideration when deciding how to travel?

____

____

____

12. On average, how many days a week do you commute to work?

	Please tick one:
1 day a week	
2 days	
3 days	
4 days	
5 days	
6 days	
7 days	

13. How far do you travel to work?

	Please tick one:
Less than a mile	
1-2 miles	
2-4 miles	
4-10 miles	
Over 10 miles	

14. Which of the following methods do you use to make the commute? (pick all that apply)

	Please tick all that apply:
Car (own)	
Car share	
Motorbike	
Bus	
Train	
Cycle	
Walk/Run	
Other (please specify)	

15. And which of these is your *most frequent* method of getting to work?

	Please tick one:
Car (own)	
Car share	
Motorbike	
Bus	
Train	
Cycle	
Walk/Run	
Other (Please specity)	

16.Why is this?

____

____

____

____

____

____

We have some questions about your neighbourhood.

17. About how long would it take to get from your home to the nearest business or facility listed below **if you walked to them**?

Please tick one

	1-5 minutes	6-10 minutes	11-20 minutes	21-30 minutes	30+ minutes	I don't know
Convenience/small grocery shop						
Supermarket						
Post Office						
Library						
Primary school						
Secondary School						
Fast food restaurant						
Coffee shop						
Bank						
Cash machine						
Chemist/Pharmacy						
GP Practice						
Hairdresser/Barber						
Newsagent						
Bus stop						
Train station						
Gym/Leisure centre						

18. Please tick the answer that best applies to you and your neighbourhood. Both local and within walking distance mean within a 10-15 minute walk from your home.

	1 strongly **disagree**	2 somewhat **disagree**	3 somewhat **agree**	4 strongly **agree**
Shops are within easy walking distance of my home.				
There are many places to go within easy walking distance of my home.				
It is easy to walk to a bus stop from my home.				
It is easy to walk to a train station from my home.				
There are pavements on most of the streets in my neighbourhood.				
My neighbourhood streets are well lit at night.				
Walkers and bikers on the streets in my neighbourhood can be easily seen by people in their homes				
There are pedestrian crossings to help walkers cross busy streets in my neighbourhood.				
There are trees along the streets in my neighbourhood.				
There are many interesting things to look at while walking in my neighbourhood.				
There is so much traffic along nearby streets that it makes it difficult or unpleasant to walk in my neighbourhood.				
The speed of traffic on most nearby streets is usually slow (30 mph or less).				
The crime rate in my neighbourhood makes it unsafe to go on walks during the day.				
The crime rate in my neighbourhood makes it unsafe to go on walks during at night.				
Parking is difficult in local shopping areas				
The streets in my neighbourhood are hilly, making my neighbourhood difficult to walk or cycle in.				

Thank you for completing this questionnaire
