# Supplementary material for: A qualitative study of active travel amongst commuters and older adults living in market towns
Source: BMC Public Health. 2023 May 10;23:840. doi: 10.1186/s12889-023-15573-3 (PMC10170734; doi:10.1186/s12889-023-15573-3)

Appendix 3: Still images from Go-along interviews

Picture 1. Cycling and walking signs

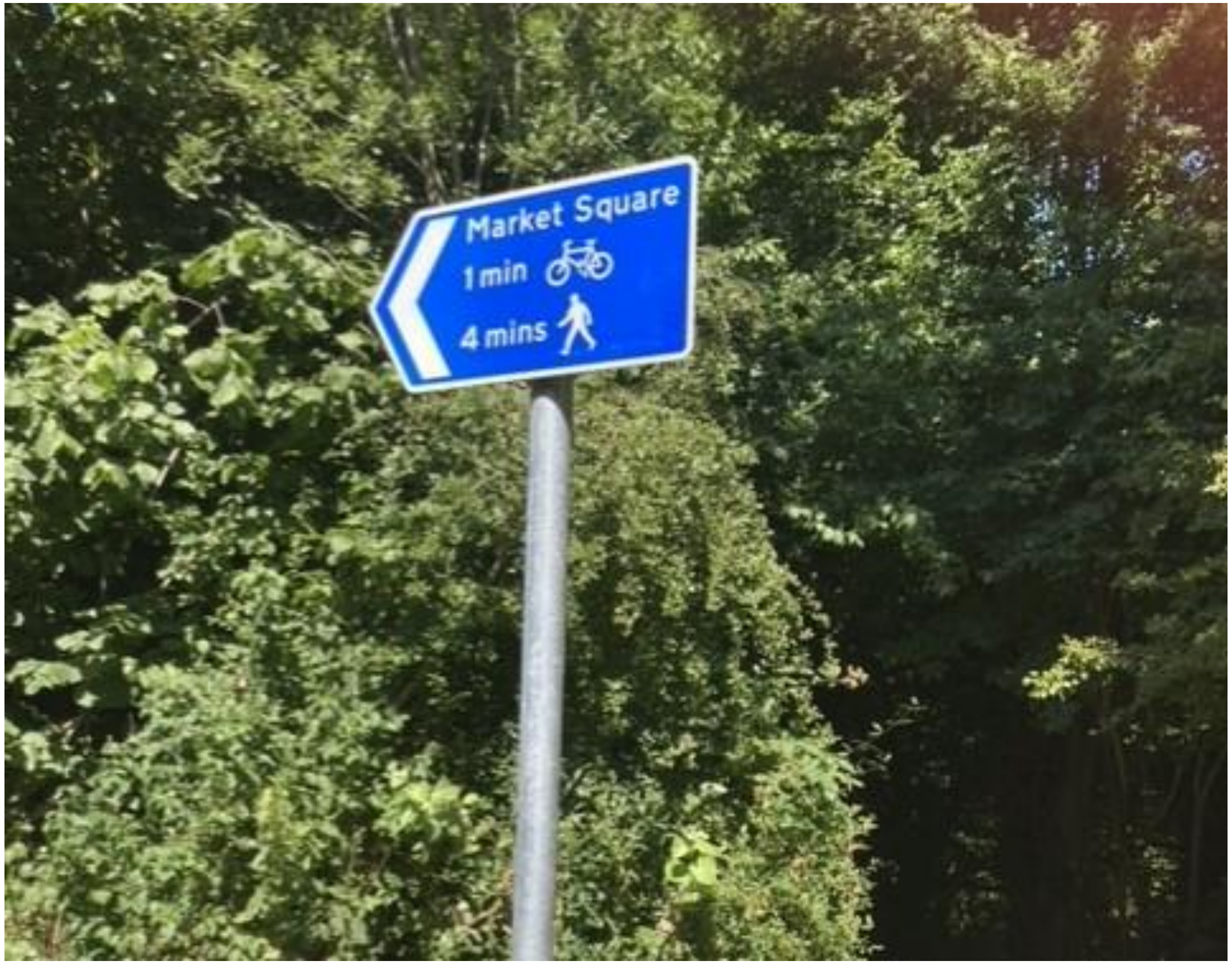

Picture 2: Shared path

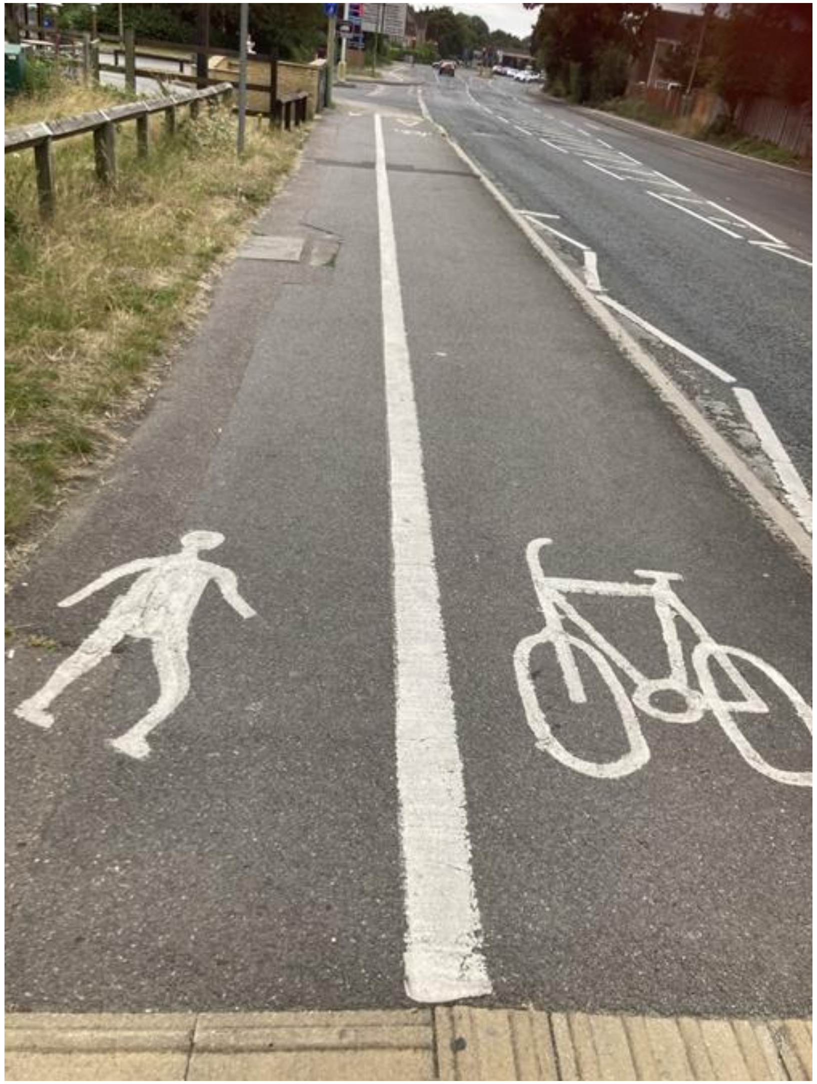

Picture 3: Overgrown and poorly lit paths

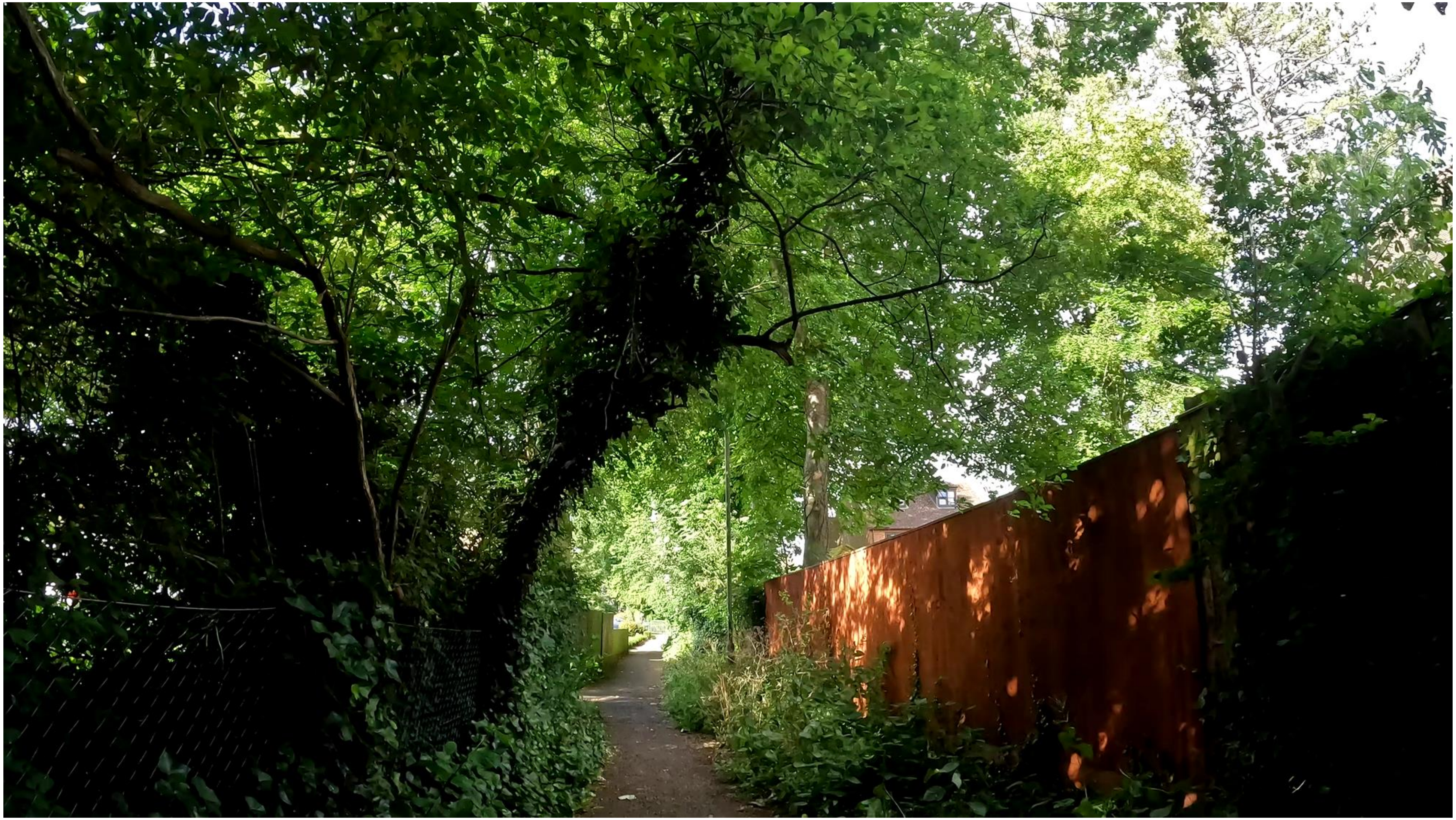

Picture 4: Poorly lit path in housing estate

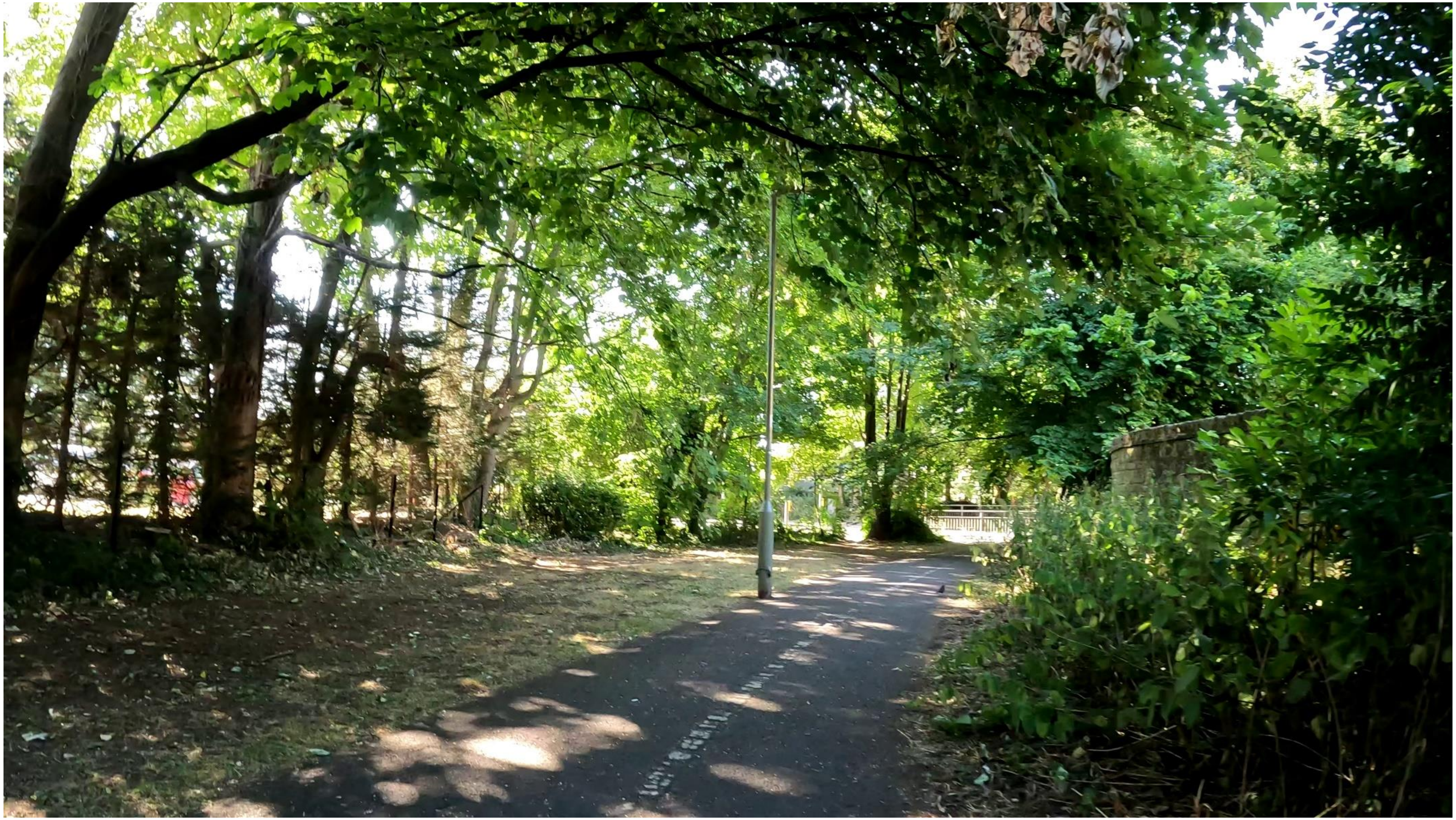

Picture 5: Overgrown shared path

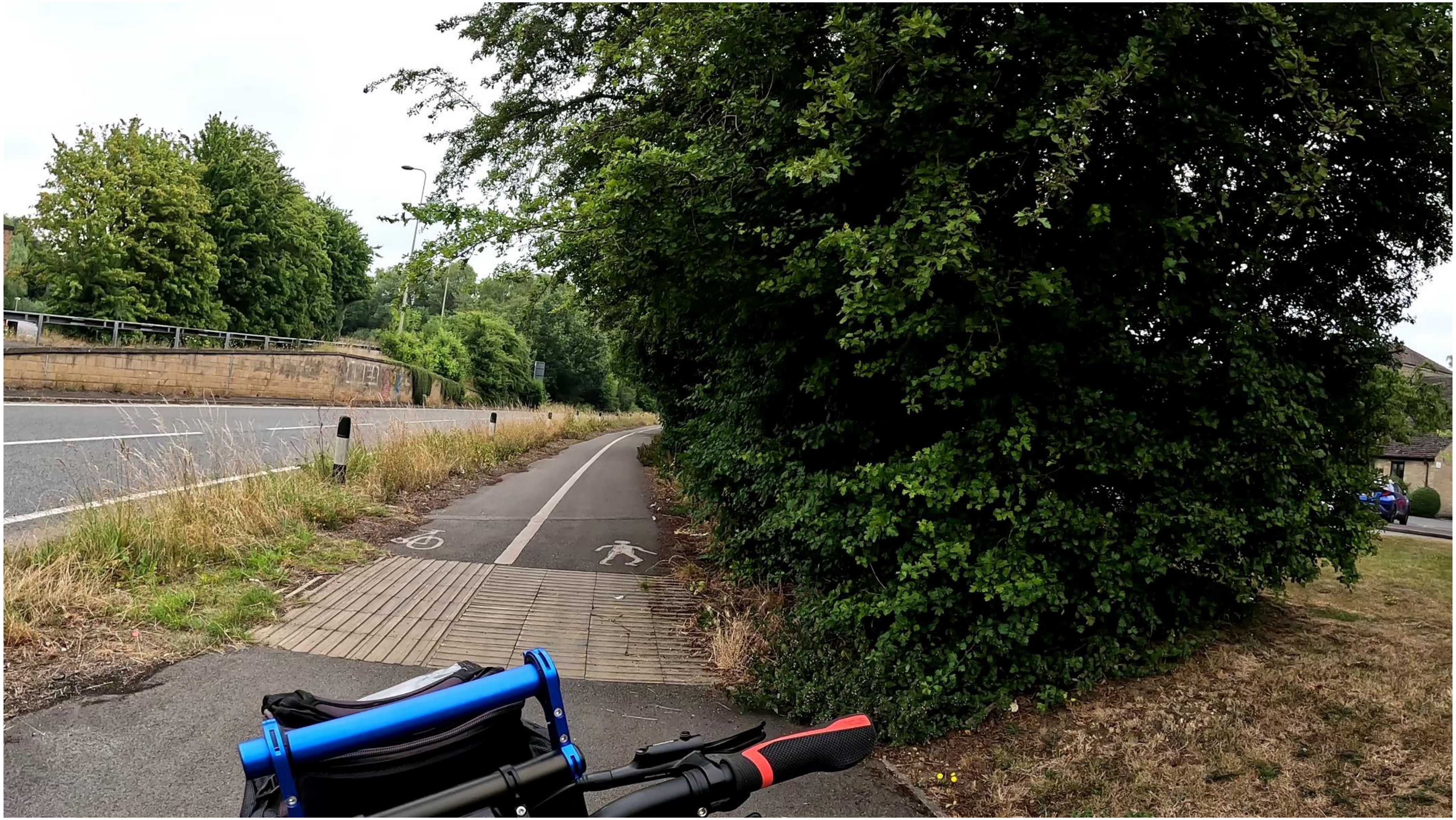

Picture 6: Long crossing times for pedestrians and cyclists

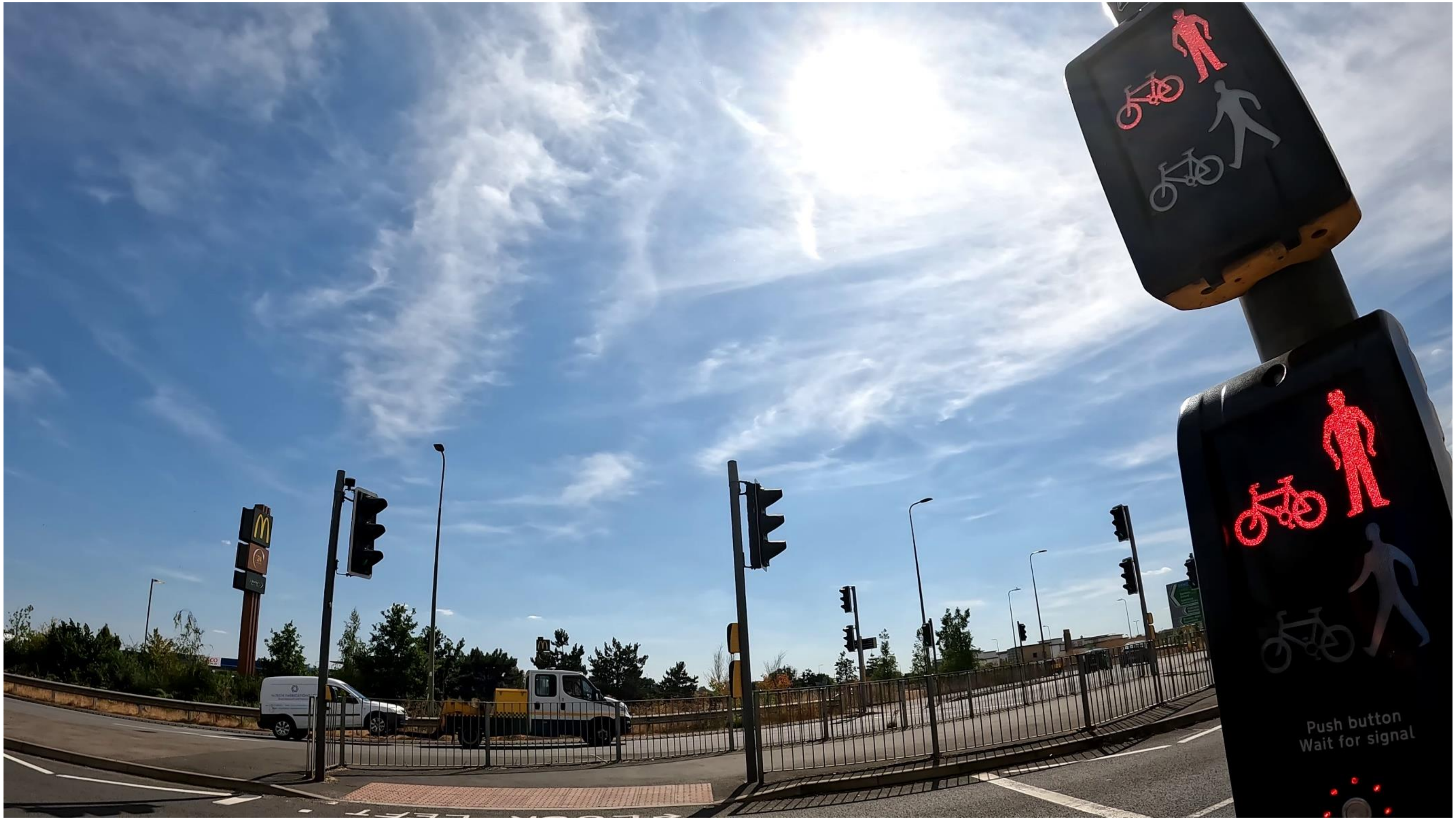

Picture 7. Unpaved track

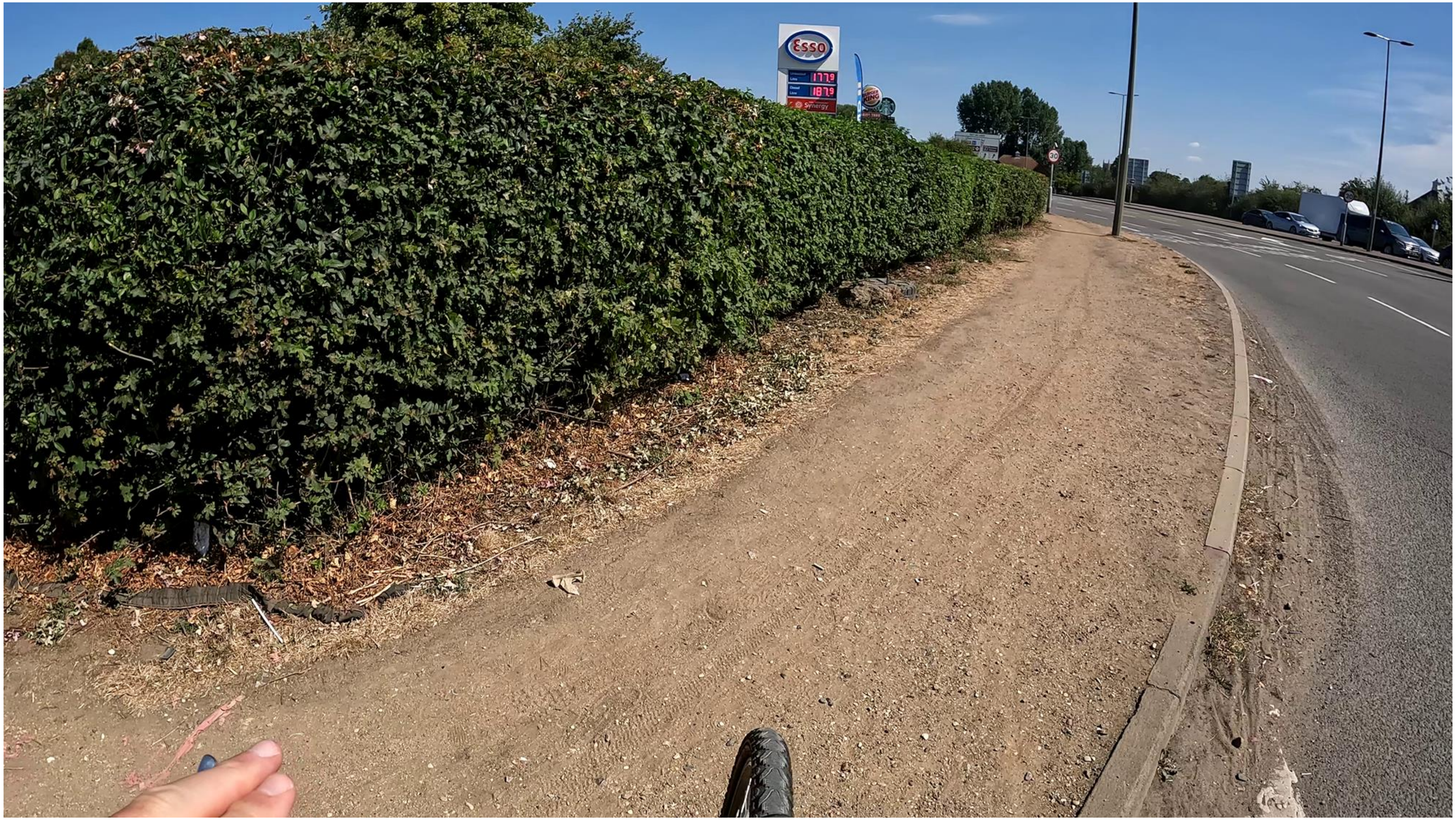

Picture 8: car parked over advisory cycle lane

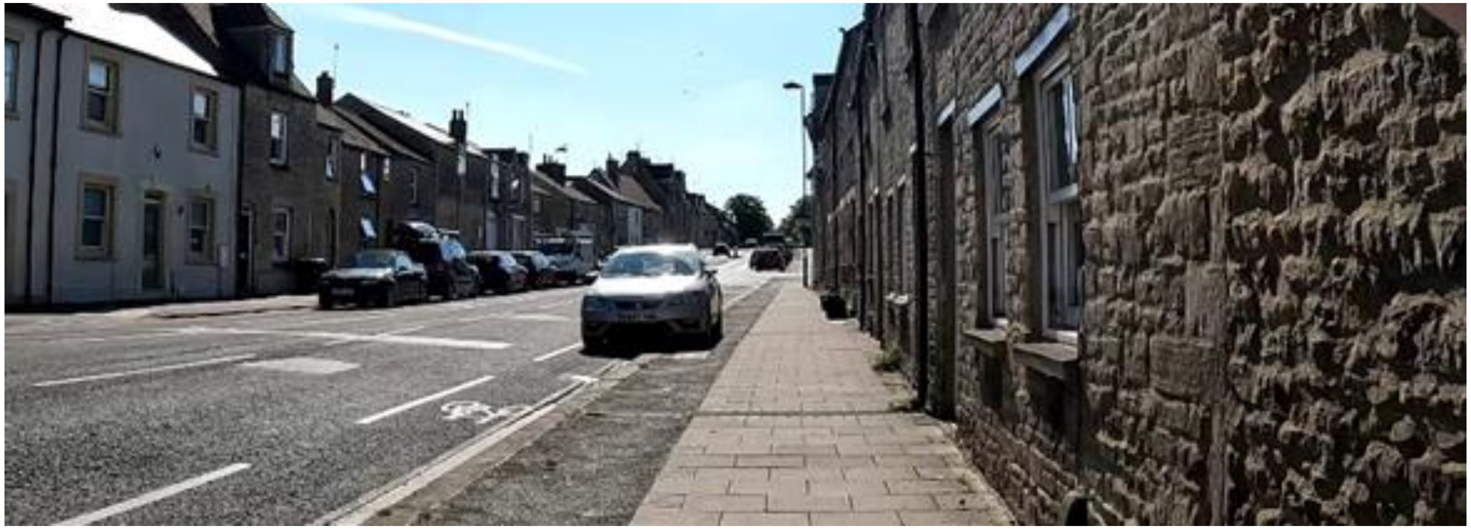

Supplement: Supplementary file 3 — Additional file 3. [file 12889_2023_15573_MOESM3_ESM.pdf]
